# High-Density Genetic Linkage Mapping of *Lepidium* Based on Genotyping-by-Sequencing SNPs and Segregating Contig Tag Haplotypes

**DOI:** 10.3389/fpls.2020.00448

**Published:** 2020-04-30

**Authors:** Mulatu Geleta, Cecilia Gustafsson, Jeffrey C. Glaubitz, Rodomiro Ortiz

**Affiliations:** ^1^Department of Plant Breeding, Swedish University of Agricultural Sciences, Alnarp, Sweden; ^2^Institute of Biotechnology, Cornell University, Ithaca, NY, United States

**Keywords:** contig tag haplotype, field cress, genetic linkage mapping, genotyping by sequencing, *Lepidium*, linkage group, single-nucleotide polymorphism

## Abstract

*Lepidium campestre* has been targeted for domestication as future oilseed and catch crop. Three hundred eighty plants comprising genotypes of *L. campestre*, *Lepidium heterophyllum*, and their interspecific F_2_ mapping population were genotyped using genotyping by sequencing (GBS), and the generated polymorphic markers were used for the construction of high-density genetic linkage map. TASSEL-GBS, a reference genome-based pipeline, was used for this analysis using a draft *L. campestre* whole genome sequence. The analysis resulted in 120,438 biallelic single-nucleotide polymorphisms (SNPs) with minor allele frequency (MAF) above 0.01. The construction of genetic linkage map was conducted using MSTMap based on phased SNPs segregating in 1:2:1 ratio for the F_2_ individuals, followed by genetic mapping of segregating contig tag haplotypes as dominant markers against the linkage map. The final linkage map consisted of eight linkage groups (*LG*s) containing 2,330 SNP markers and spanned 881 Kosambi cM. Contigs (10,302) were genetically mapped to the eight *LG*s, which were assembled into pseudomolecules that covered a total of ∼120.6 Mbp. The final size of the pseudomolecules ranged from 9.4 Mbp (*LG-4*) to 20.4 Mpb (*LG-7*). The following major correspondence between the eight Lepidium *LG*s (*LG-1* to *LG-8*) and the five *Arabidopsis thaliana* (*At*) chromosomes (*Atx-1*–*Atx-5*) was revealed through comparative genomics analysis: *LG-1&2*_*Atx-1*, *LG-3*_*Atx*-*2*&*3*, *LG-4*_*Atx-2*, *LG-5*_*Atx-2*&*Atx-3*, *LG-6*_*Atx-4*&*5*, *LG-7*_*Atx-4*, and *LG-8*_*Atx-5*. This analysis revealed that at least 66% of the sequences of the *LG*s showed high collinearity with *At* chromosomes. The sequence identity between the corresponding regions of the *LG*s and *At* chromosomes ranged from 80.6% (*LG-6*) to 86.4% (*LG-8*) with overall mean of 82.9%. The map positions on *Lepidium LGs* of the homologs of 24 genes that regulate various traits in *A. thaliana* were also identified. The eight *LG*s revealed in this study confirm the previously reported (1) haploid chromosome number of eight in *L. campestre* and *L. heterophyllum* and (2) chromosomal fusion, translocation, and inversion events during the evolution of *n* = 8 karyotype in ancestral species shared by *Lepidium* and *Arabidopsis* to *n* = 5 karyotype in *A. thaliana*. This study generated highly useful genomic tools and resources for *Lepidium* that can be used to accelerate its domestication.

## Introduction

Today’s major crop species are the results of thousands of years of intentional and unintentional selection of traits that brought genetically determined changes in the ancestral wild plant species (Burger et al., 2008). Domestication of a crop species is generally a very slow and long-term process that leads to significant changes in major traits that are regarded as “domestication syndrome” traits, such as determinate growth habit, increased seed size, loss of seed dormancy, and reduced pod shattering (Harlan et al., 1973; Doebley et al., 2006; Weeden, 2007; Burger et al., 2008). However, progress in genomic research that include comparative genomics, gene identification, annotation of whole genome sequences (WGS), development of genome-wide molecular markers, and genome-wide association studies (GWAS) for various crops led to deep insight into the process of plant domestication and evolution (Geleta and Ortiz, 2016). The use of genomic tools and resources in combination with conventional plant breeding methods is becoming essential in the development of new crop cultivars in a relatively shorter time than before (Pérez-de-Castro et al., 2012). Genomic tools and resources, such as a variety of molecular markers, high-density genetic linkage, quantitative trait locus (QTL), and genome-wide association maps are becoming the cornerstone of plant breeding, as they facilitate marker-assisted and genomic selection (Collard and Mackill, 2008; Lorenz et al., 2012; Lopez-Cruz et al., 2015; Qu et al., 2017; Koech et al., 2019). Through the use of these tools and resources and the analyses of the genetics of “domestication syndrome” traits, a good insight into the evolutionary changes that have occurred during plant domestication have been gained and can be used to facilitate a rapid domestication of new plant species.

*Lepidium* L. is a large, undomesticated genus in the Brassicaceae family that comprises 231 species distributed around the world (Al-Shehbaz et al., 2006). Indigenous *Lepidium* species are found on all continents (Al-Shehbaz, 1986), and they often grow in habitats with less competition for resources and space, such as roadsides, railway sides, and disturbed areas. *L. campestre* (L.) R. Br. (field cress), an annual or biennial (Mulligan, 1961) diploid species with 2*n* = 2*x* = 16 chromosomes (Rice et al., 2015), has wide distribution in Europe including Nordic countries. Based on its various desirable characteristics including winter hardiness, promising potential for high seed yield, self-compatibility, synchronous seed maturity, and suitability as an undersown catch crop (Al-Shehbaz, 1986; Merker and Nilsson, 1995; Eriksson, 2009; Geleta et al., 2014), it has been considered for domestication as a future oilseed and catch crop in Sweden to contribute to increased global production of vegetable oil as well as diversification of agroecosystems. Although the oil content of *L. campestre* was initially reported to be ∼20% (Nilsson et al., 1998), further studies of a wide collection of European and North American *L. campestre* accessions showed that the oil content varies from 12 to 20% (Mulatu Geleta et al., SLU, unpublished data). Its seed oil is mainly composed of linolenic acid (C18:3; 34–39%), erucic acid (C22:1; 22–25%), oleic acid (C18:1; 12–16%), and linoleic acid (C18:2; 8–11%).

Research and breeding activities of varying intensity have been ongoing during the last 25 years at the Swedish University of Agricultural Sciences (SLU), contributing to the domestication of *L. campestre* (Merker and Nilsson, 1995; Andersson et al., 1999; Börjesdotter, 1999; Eriksson, 2009; Merker et al., 2010; Ivarson et al., 2013; Geleta et al., 2014; Ivarson et al., 2016; Gustafsson et al., 2018). Domestication of *L. campestre* should result in significant improvement in various major traits, such as oil content and quality, pod shatter resistance, and seed yield (Eriksson, 2009; Geleta et al., 2014). For use as edible oil, antinutritional compounds, such as glucosinolates and erucic acid, will have to be eliminated or drastically reduced through breeding (Andersson et al., 1999; Ivarson et al., 2016).

Given that perenniality is a favorable trait in catch crops, the overall goal of the domestication of *L. campestre* is to develop both biennial and perennial cultivars. However, developing perennial *L. campestre* requires its interspecific hybridization with perennial *Lepidium* species, such as *L. heterophyllum* Benth. and *Lepidium hirtum* (L.) Sm. (Mulligan, 1961; Mummenhoff et al., 1995). Both species are closely related to *L. campestre* (Mummenhoff et al., 2001; Lee et al., 2002) and share the same ploidy level and chromosome number (2*n* = 2*x* = 16) (Rice et al., 2015). The interspecific hybridization of *L. campestre* with these two species was successful, and perennial breeding lines derived from these hybrids have been developed (Mulatu Geleta et al., SLU, unpublished data). In addition to the perenniality trait, hybridization between these species led to a larger variation in various desirable traits as compared to the variation within either of the parental species, providing wider opportunities for further breeding. This study was conducted to develop genomic tools and resources for *Lepidium* in order to understand its genome as well as accelerate its domestication.

## Materials and Methods

### Plant Material

A total of 380 plants that comprises three genotypes of *L. campestre* (*Par_1*, *Stu_7* and *C92_2_3*), two genotypes of *L. heterophyllum* (*Par_2* and *Hast_*3), and a mapping population of 375 F_2_ plants derived from interspecific hybrid of *Par_1* and *Par_2* (parents) were used in this study. *Par_1* and *Stu_7* were genotypes collected from Arlid (unknown exact location) and Stuvsta (59°15′25″ N, 17°58′51″ E), Sweden, respectively. *C92_2_3* was a genotype sampled from IPK (Germany) accession *LEP-92* originally collected from Greece. *Par_2* was a genotype that belongs to a US Department of Agriculture (USDA)-Agricultural Research Service (ARS) accession *LH 597856* originally collected from Spain, whereas *Hast_3* is a genotype collected from Hästveda, Sweden (56°17′21″ N, 13°56′09″ E). Genomic data from the mapping population and the two parents were used for genetic linkage mapping and various statistical analyses. The two *L. campestre* and one *L. heterophyllum* genotypes were included to estimate various genetic diversity parameters within and among the two *Lepidium* species.

### DNA Extraction

Seeds from target samples were planted in a greenhouse at the Department of Plant Breeding, SLU, Alnarp, Sweden. Young leaf tissue was sampled in Eppendorf tubes from individual plants and immediately frozen in liquid nitrogen, and genomic DNA was extracted as described in Gustafsson et al. (2018). The quality and quantity of the extracted DNA was assessed using 1% agarose gel electrophoresis and a NanoDrop^®^ ND-1000 Spectrophotometer (Saveen Warner, Sweden). The extracted DNA samples were then sent to the Genomic Diversity Facility, Cornell University, USA, for genotyping by sequencing (GBS).

### GBS Optimization and Analysis

A number of restriction enzymes were tested to determine the best enzyme that produces fragment size distribution suitable for the construction of GBS library. At the end, *Ape*KI (G^∗^CWGC), a 4-base cutter enzyme was selected, as majority of fragments produced were < 500 bp and hence were appropriate for Illumina sequencing. The reference genome-based pipeline TASSEL-GBS (Glaubitz et al., 2014) was used for this GBS analysis, where a draft whole genome sequence of *L. campestre* assembled in-house was used as a reference genome. In this GBS analysis, a total of 7,591,461 tags were generated after merging, and these tags were aligned against the in-house assembled *L. campestre* genome. Of these tags (3,688,674 tags), 48.6% were uniquely aligned to the reference genome, whereas 8.8 and 42.6% were multiply aligned and unaligned, respectively. SNP calling after aligning the tags to the reference genome resulted in 165,892 SNPs. VCFtools version v0.1.12a (Danecek et al., 2011) was used to calculate heterozygosity as well as the depth and missingness of the resultant SNPs. Filtering out of SNPs with minor allele frequency (MAF) below 0.01 and missing data per site above 90% resulted in 126,859 SNPs, of which 120,438 were biallelic. PLINK version v1.07 was used to generate a multidimension scaling (MDS) plot based on the 120,438 genome-wide biallelic SNPs.

### Linkage Map Construction With GBS SNPs

The 126,859 GBS SNP markers were processed using VCFtools version 0.1.15 (Danecek et al., 2011) in the following order: (1) only SNPs with MAF of at least 40% were retained; (2) genotypes supported by a read depth of less than seven were set to missing; (3) SNPs with more than 10% missing data were discarded; (4) SNPs deviating from 1:2:1 segregation with *p* < 0.01 were discarded; (5) the SNPs were thinned so that no two SNPs were <65 bases apart (i.e., only one SNP was retained per 64-base GBS tag locus); and (6) the genotypes were converted to a numerical format to facilitate further processing in R (R Core Team, 2016). The genotypes were then phased based upon the two parents, and the resultant 2,352 phased SNPs for the 375 F_2_ individuals were reformatted for input into MSTMap (Wu et al., 2008) with a custom R script. The parameters used for linkage map construction with MSTMap include Kosambi distance with cutoff *P-*value of 1e^–^^12^, number of map distance of 10, number of map size of 1, and missing threshold of 0.15.

The resulting linkage map of eight linkage groups was reformatted for loading into R with a custom awk command, and then, a custom R script was used to merge it with the genotypic data and count the number of crossovers per individual across the eight linkage groups. Nine outlier individuals with more than 66 crossovers were discarded from further analysis, and a new linkage map was constructed with MSTMap using the remaining 366 individuals (same parameters as above). Twenty-one outlier SNPs with more than 15 double crossovers were then identified using R and excluded, and a final linkage map was constructed with MSTMap for the remaining 2,331 SNPs (again with the same parameters). The R package R/qtl (Broman et al., 2003) was then used to correct genotyping errors and impute most of the missing genotypes [using the fill.geno() function with method = “maxmarginal,” map.function = “kosambi,” and min.prob = 0.8]. The resulting genotypes were visualized with a custom R script (Supplementary Figure S1) and output in R/qtl csvr format to facilitate conversion to hapmap format via a custom awk command.

### Genetic Mapping of Segregating GBS Tag Sequences

Custom Tassel3 (Bradbury et al., 2007) code was used to filter the TagsOnPhysicalMap (TOPM) and TagsByTaxa (TBT) data structures produced in the GBS SNP calling pipeline (Glaubitz et al., 2014) so that they contained only tags with unique alignment positions with no sequence divergence from the contig-level reference assembly. The TBT was also filtered to retain only the F_2_ individuals present in the final linkage map and only tags that appeared to segregate in the F_2_ [present in at least 30 and no more than 256 (80%) of the 366 individuals]. Each tag was then genetically mapped as a dominant marker against the linkage map. Because GBS was performed at low sequencing depth, the absence of a tag in an F_2_ individual is not always informative; hence, only the progeny in which a given tag was observed were used to calculate the recombination rate between that tag and each SNP in the linkage map. For this subset of progeny, the recombination rate was calculated as: min[nHomPar_2/(nHomPar_1 + nHomPar_2), nHomPar_1/(nHomPar1 + nHomPar2)] where nHomPar_1 was the number of individuals with the tag that were homozygous for the parent_1 allele at the SNP in question, and nHomPar_2 was the number of individuals with the tag that were homozygous for the parent_2 allele. Heterozygous individuals at the SNP were excluded as non-informative. Tags were considered genetically mapped if the recombination rate was < 5%, and the sample size (nHomPar1 + nHomPar2) was at least 30 (two-tailed binomial *P* < 6e^–^^8^). The genetic mapping span (GeneticStart to GeneticEnd) for a tag was from the first to last SNP on the linkage group with the same minimum recombination rate, and the genetic mapping position (GeneticMean) was the mean of this span, where the SNP positions were numbered consecutively from 1 to the number of SNPs on the linkage group.

### Genetic Mapping of Contig Tag Haplotypes and Assembly Into Pseudomolecules

In light of the low sequencing depth of GBS, the statistical power for mapping contigs was increased by combining all of the concordant genetically mapped tags for a contig into a single tag haplotype, with the presence of any given tag imputing the presence of the contig haplotype as a whole. For contigs with multiple genetically mapped tags, the consensus linkage group assignment was determined by a majority rule weighted by sample size. For those tags mapped to the same linkage group, the consensus genetic mapping position was determined as the sample size weighted average of the tag mapping positions (GeneticMean). Tags with genetic mapping positions on the same linkage group and within 50 SNPs of the consensus genetic mapping position were considered as agreeing with the consensus and were thus merged into a contig tag haplotype, by summing their tag counts across each taxon. The contig tag haplotypes were then genetically mapped in the same manner as the individual tags, except that a minimum recombination rate of 10% was used, and to prevent false positives from occasional sequencing errors, a contig tag haplotype was only considered present in a genotype if the count for that genotype was at least 5% of the mean of the non-zero counts across genotypes.

The genetically mapped contigs were ordered into pseudomolecules according to the following rules, using custom awk and bash commands: (1) contigs placed on the linkage map via segregating SNPs were kept in the same relative order, regardless of whether they were mapped by tag haplotype or not, in which (a) contigs with multiple mapped SNPs were quasi-oriented if the centimorgan position differed between the first and last SNP in the contig, with the first and last SNP defined by physical position in the contig, (b) a contig with multiple SNPs was not always contiguous in the map, as it was sometimes comingled with one or more additional contigs, and the order of comingled contigs was resolved by the position of their first SNP in the linkage map, and (c) contigs without genetic consensus (e.g., with an equal number of SNPs mapping to different linkage groups) were removed from the assembly; (2) contigs mapped only by tag haplotype were placed immediately after the contig containing their GeneticMean SNP, with the following sort order: GeneticStart, GeneticEnd, contig type (scaffold < contig), contig name; (3) ordered contigs on a linkage group/pseudomolecule were separated by 100 N’s; and (4) the linkage groups/pseudomolecules were named *LG-1*, *LG-2*, *LG-3*, *LG-4*, *LG-5*, *LG-6*, *LG-7*, and *LG-8*, by modifying the names of the linkage groups assigned by the MSTMap software.

## Results

### Multidimensional Scaling, Heterozygosity, and Inbreeding Coefficient

**FIGURE 1:**
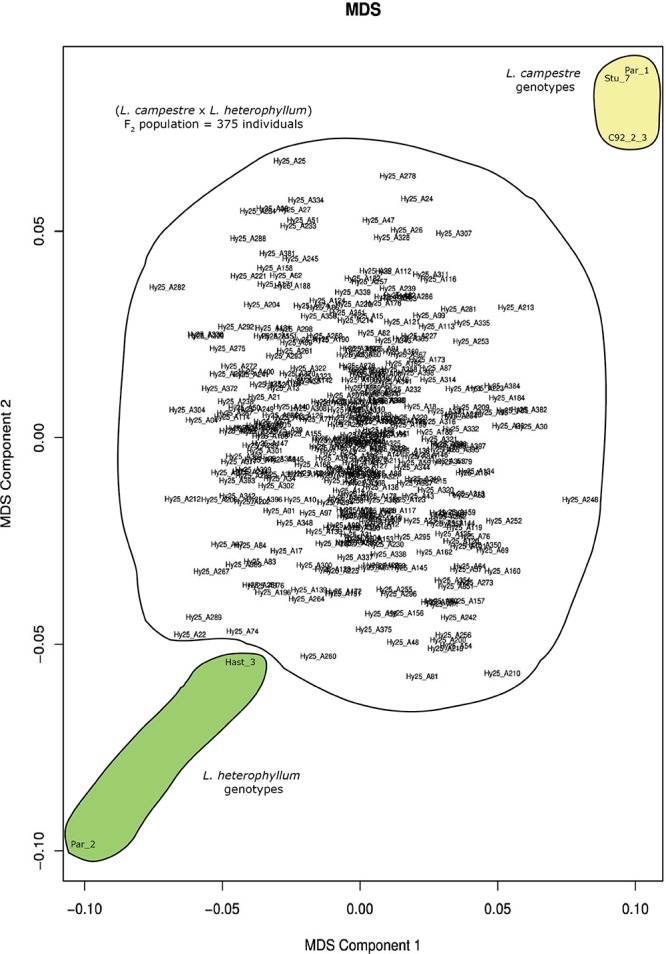
Multidimension scaling (MDS) plot generated for 380 individual plants comprising three *L. campestre* and two *L. heterophyllum* genotypes as well as 375 F_2_ mapping population based on 120,438 genome-wide biallelic SNPs.

Ninety-five percent (120,438) of the 126,859 filtered SNPs were biallelic. Multidimensional scaling based on these genome-wide biallelic SNPs displayed the distribution of the 375 individuals of the mapping population, their parents, as well as the other three genotypes included in the study (Figure 1). In this analysis the two parents, *Par_1* and *Par_2*, were positioned at the top right and bottom left corners of the plot, respectively, and the MDS clearly displayed the clustering of the three *L. campestre* genotypes (*Par_1*, *Stu_7* and *C92_2_3*) and the two *L. heterophyllum* genotypes (*Par_2* and *Hast_3*) at their respective corners (Figure 1). The two Swedish genotypes of *L. campestre* (*Par_1* and *Stu_7*) were more closely related to each other than to *L. campestre* genotype originally collected from Greece (*C92_2-3*). The F_2_ individuals spread widely across the two dimensions with the highest concentration around the center (Figure 1). The distribution of these individuals shows that they represented the whole F_2_ population very well. Based on these data, it is possible to select individuals that are genetically more similar to *L. campestre* (the target species for domestication) for further breeding. For example, individuals such as *Hy25_A24* and *Hy25_A307* would be among the top candidates for further breeding or crossbreeding with *L. campestre*, if they have desirable traits such as perenniality.

**FIGURE 2:**
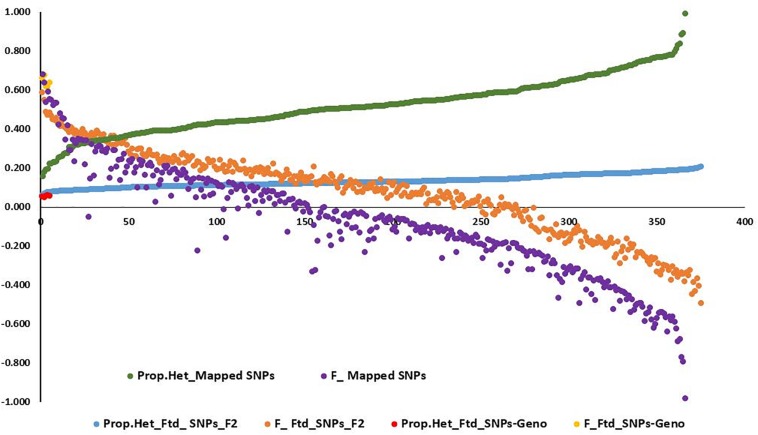
Graph showing the distribution of the proportion of heterozygosity at (1) mapped single-nucleotide polymorphism (SNP) loci for the 375 F_2_ population (green) and their corresponding inbreeding coefficient (purple), (2) filtered SNP loci for the 375 F_2_ population (blue) and their corresponding inbreeding coefficient (orange), and (3) filtered SNP loci for the five genotypes (three *L. campestre* and two *L. heterophyllum*) (red) and their corresponding inbreeding coefficient (yellow).

Observed heterozygosity (*Ho*) and expected heterozygosity (*He*) as well as inbreeding coefficient (*F*) were calculated for the three *L. campestre*, two *L. heterophyllum* samples, as well as for of the F_2_ individuals across thousands of SNP loci (2,331–100,760 loci) (Table 1 and Figure 2). These parameters were calculated for each individual after removing loci with missing data. In the case of all filtered SNPs (126,859), 50,439–10,076 SNP loci remained per individual after removing the loci with missing data. Similarly, removing the loci with missing data for mapped SNPs resulted in 2,326–2331 loci per individual. For the three *L. campestre* genotypes, only 5.1–5.7% of the loci were heterozygous, and the mean heterozygosity was 5.4% (*P_Ho* = 0.054; Table 1). Similarly, heterozygous loci accounted for a mean of 5.5% in *L. heterophyllum*. Inbreeding coefficient (*F*) was 0.64 on average for both species. In the case of F_2_ population, the proportion of observed heterozygosity was 13.1 and 51.9% for all filtered and mapped SNPs, with corresponding inbreeding coefficient of 0.07 and −0.08, respectively (Table 1 and Figure 2).

**TABLE 1:** Number of SNP loci (*NL*), number of observed heterozygosity (*N_H_o_*), number of expected heterozygosity (*N_H_e_*), proportion of observed heterozygosity (*P_H_o_*), and inbreeding coefficient (*F*) for the three *L. campestre*, two *L. heterophyllum* genotypes, and the *F*_2_ mapping population.

**Genotype/Population**	***NL***	***N_H_o_***	***N_H_*e*_***	***P_H_*o*_***	***F***
Par_1^*a*^	73,053	4,149	10,909	0.057	0.620
C92_2_3^*a*^	66,448	3,396	9,976	0.051	0.660
Stu_7^*a*^	67,034	3,631	9,974	0.054	0.636
*L. c_* Mean^a^*	68,845	3,725	10,286	0.054	0.639
Par_2^*b*^	67,360	3,958	10,286	0.059	0.615
Hast_3^*b*^	50,439	2,524	7,742	0.050	0.674
*L. h_* Mean^b^**	58,900	3,241	9,014	0.055	0.645
F2_375_Min^*c*^	72,435	4,293	10,420	0.059	0.588
F2_375_Max^*c*^	96,692	19,241	14,063	0.199	–0.368
F2_375_Mean^*c*^	85,885	11,280	12,103	0.131	0.072
F2_366_Min^*d*^	2,331	364	1,140	0.156	0.681
F2_366_Max^*d*^	2,331	2,306	1,166	0.989	–0.979
F2_366_Mean^*d*^	2,331	1,209	1,119	0.519	–0.079

*NL is different for different individuals because loci with missing data for each individual were excluded.*

*^a^L. campestre.*

*^b^L. heterophyllum.*

*^*c*^Minimum, maximum, and mean number of SNP loci per individual and corresponding values for the other four parameters among the 375 F_2_ individuals.*

^d^Minimum, maximum, and mean number of SNP loci per individual and corresponding values for the other four parameters among the 366 F_2_ individuals used in the genetic linkage mapping, calculated based on mapped SNPs.

*Mean values for L. campestre genotypes.

**Mean values for L. heterophyllum genotypes.

### Linkage Map Construction With GBS SNPs

**FIGURE 3:**
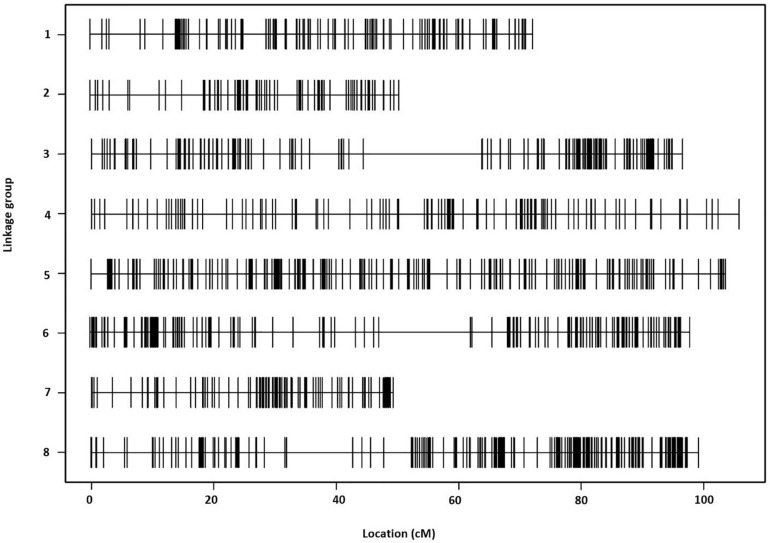
The *Lepidium* linkage map comprising eight linkage groups and showing the distribution of 2,330 single-nucleotide polymorphism (SNP) markers across a span of 881 Kosambi cM in total.

**FIGURE 4:**
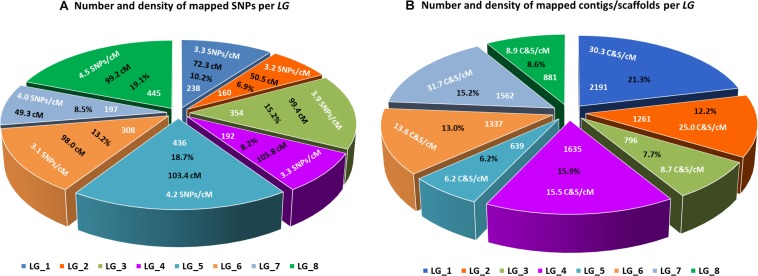
*Lepidium* linkage groups. **(A)** Size of each linkage group in centimorgans (cM), number and percentage of SNPs mapped to each linkage group, and average number of mapped SNPs per centimorgan. **(B)** Number and percentage of contigs/scaffolds mapped to each linkage group, and average number of mapped contigs/scaffolds per centimorgan.

**FIGURE 5:**
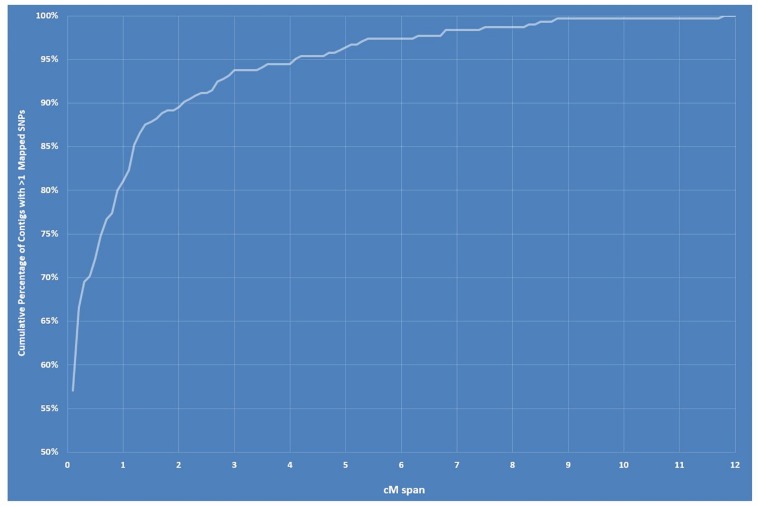
Graph showing cumulative percentage of contigs/scaffolds with > 1 mapped single-nucleotide polymorphisms (SNPs) within a centimorgan span between 0 and 12.

The final linkage map consisted of eight linkage groups (*LG*s) containing 2,331 SNP markers derived from 1,044 contigs, and spanned 881 Kosambi cM in total (Figure 3, see also Supplementary Figure S1). The number of mapped SNPs per contig varied from 1 to 66, and 305 (29.2%) of the 1,044 contigs contained more than one mapped SNPs (Supplementary Tables S1, S2). Of the 305 contigs possessing more than one mapped SNPs, only one was mapped to more than one linkage groups (scaffold140, with four SNPs on *LG-2* and one SNP on *LG-5*). The SNP on *LG-5* was excluded, and hence, the total number of SNPs shown on the linkage map is 2,330 (Figure 4 and Supplementary Tables S1, S2). The SNPs on each contig were mapped within 1 cM of each other for 81.0% of the contigs, within 5 cM for 96.4%, and within 10 cM for 99.7% (Figure 5 and Supplementary Tables S2). These results indicate that the assembly of paired end reads into contigs was highly accurate. The number of SNPs mapped to *LG-1* to *LG-8* were 238 (10.2%), 160 (6.9%), 354 (15.2%), 192 (8.2%), 436 (18.7%), 308 (13.2%), 197 (8.5%), and 445 (19.1%), respectively. These SNPs spanned 72.3, 50.5, 99.4, 105.8, 103.4, 98.0, 49.3, and 99.2 cM and hence have an average SNP density of 3.3, 3.2, 3.9, 3.3, 4.2, 3.1, 4.0, and 4.5 SNPs/cM for *LG-1* to *LG-8* in that order (Figure 4).

### Genetic Mapping of Segregating GBS Tag Sequences and Contig Tag Haplotypes

In total, 34,342 segregating 64-base GBS tag sequences from 9,943 contigs were placed on the genetic map within a recombination fraction of 5% of one or more SNP markers (Supplementary Tables S3). Of these tags, 33,832 either agreed with the genetic consensus for their respective contig or were the sole representative tag, whereas 510 (1.5%) disagreed with the consensus and were excluded from further analysis, along with 12 contigs that did not display genetic consensus. The remaining 9,931 contigs with genetic consensus were all successfully mapped, as a segregating tag haplotype, to within a recombination fraction of 7.9% of one or more SNPs in the linkage map (Supplementary Tables S3). In total, 10,302 contigs were genetically mapped, with 371 mapped only by SNP, 673 mapped by both SNP and contig tag haplotype, and 9,258 mapped only by contig tag haplotype (Supplementary Tables S1). The sequences of the 10,302 mapped contigs have been deposited at DDBJ/ENA/GenBank as Whole Genome Shotgun project under the accession number WJSH00000000. These contigs were assembled into a pseudomolecule fasta file according to the ordering and orientation rules described in section Materials and Methods above. The pseudomolecules in the assembly covered 120,594,250 bases (∼120.6 Mbp) (Table 2), of which 116,577,053 (96.7%; ∼116.6 Mbp) were not N. The final size of the *LG*s ranged from 9.4 to 20.4 Mbp with *LG-1 to LG-8* having a size of 18.9, 10.1, 13.1, 9.4, 15.2, 18.7, 20.4, and 14.9 Mbp (Table 2), and accounted for 15.6, 8.3, 10.9, 7.8, 12.6, 15.5, 16.9, and 12.3% of the 120.6 Mbp total assembled sequences, in that order. The G + C content of the *LG*s ranged from 34.9 (*LG-4*) to 35.9 (*LG-3*) with a mean of 35.5% (Table 2).

**TABLE 2:** The assembled size of the eight *Lepidium* linkage groups (*LG*s) in megabase pair and the homeology, sequence identity, and length of their sequences corresponding to *A*. *thaliana* chromosome (*Atx*) sequences.

		**Sequence**		**Max**	**Total**	**Query coverage**	**Identity**	**Number of**	**LMSL^*c*^**
***LG***	**%G + C**	**size (Mbp)**	***Atx***	**score**	**score**	**(%)**	**(%)**	**matches**	**(in kbp)**
*LG-1*	35.4	18.85	1	5,381	4,499,000	21	92	14,565	3.8
			2	2,618	473,900	2	84	2,961	
			3	2,458	598,600	2	83	3,744	
			4	2,356	389,500	2	81	2,874	
			5	1,936	492,600	2	79	3,599	
			Total/mean	–	–	29^*a*^	83.8^*b*^	–	
*LG-2*	35.4	10.07	1	4,082	2,458,000	14	84	8,478	3.8
			5	1,920	490,100	3	78	2,779	
			2	2,849	211,100	2	87	1,286	
			3	3,442	301,800	2	84	1,726	
			4	1,999	280,900	2	79	1,662	
			Total/mean	–	–	23^*a*^	82.4^*b*^	–	
*LG-3*	35.9	13.11	3	19,392	3,793,000	26	91	7,917	13.8
			2	11,250	1,517,000	11	88	4,198	
			5	4,247	800,500	6	80	4,186	
			4	3,255	482,800	4	80	2,689	
			1	4,816	600,600	4	86	4,080	
			Total/mean	–	–	51^*a*^	85.0^*b*^	–	
*LG-4*	34.9	9.44	2	6,945	2,308,000	25	84	6,793	5.6
			1	2,572	829,000	5	82	4,538	
			3	2,444	566,700	4	77	3,288	
			5	7,954	473,800	4	91	2,836	
			4	2,375	319,900	3	80	1,913	
			Total/mean	–	–	41^*a*^	82.8^*b*^	–	
*LG-5*	35.6	15.22	3	15,324	4,570,000	25	85	9,820	13.8
			2	6,082	916,900	6	81	3,732	
			5	4,228	919,400	5	83	4,839	
			1	4,452	699,800	3	84	4,589	
			4	5,039	359,900	2	85	2,409	
			Total/mean	–	–	41^*a*^	83.6^*b*^	–	
*LG-6*	35.5	18.66	4	12,103	2,441,000	10	84	7,171	11.1
			5	5,649	1,919,000	9	84	6,572	6.7
			1	2,967	747,700	3	85	5,007	
			2	1,996	346,000	2	79	2,458	
			3	2,379	560,400	2	74	3,633	
			Total/mean	–	–	26^*a*^	81.2^*b*^	–	
*LG-7*	35.8	20.38	4	14,077	4,092,000	18	82	10,561	14.1
			5	3,141	1,408,000	5	79	6,558	
			1	2,562	821,000	3	81	5,335	
			2	3,162	599,300	3	79	3,256	
			3	4,124	633,700	2	82	3,874	
			Total/mean	–	–	31^*a*^	80.6^*b*^	–	
*LG-8*	35.4	14.85	5	12,510	6,087,000	39	82	11,383	12.8
			3	3,926	832,100	6	85	4,343	
			1	3,927	609,800	3	78	4,232	
			2	2,825	369,400	2	86	2,538	
			4	6,042	366,900	2	86	2,445	
			Total/mean	–	–	52^*a*^	83.4^*b*^	–	

*The eight linkage groups comprise 10,302 contigs/scaffolds with a total sequence length of 120.59 Mbp. The accession numbers and sequence size (bp) of A. thaliana chromosomes used in this analysis, respectively, are as follows: chromosome_1 (NC_003070.9, 30427671), chromosome_2 (NC_003071.7, 19698289), chromosome_3 (NC_003074.8, 23459830), chromosome_4 (NC_003075.7, 18585056), and chromosome_5 (NC_003076.8, 26975502). The mean G + C content of the LGs is 35.5%; the overall mean sequence identity of the LGs with At chromosomes was 82.9%; E-values of all hits are 0.0.*

*^*a*^Total.*

*^*b*^Mean.*

*^*c*^LMSL, longest aligned sequence length between the corresponding LGs and Atxs.*

### Comparative Analysis of *Lepidium* Linkage Groups and *Arabidopsis thaliana* Chromosomes

Basic Local Alignment Search Tool (BLAST)^1^ was used to search *Arabidopsis thaliana* genome (taxid:3702) at the GenBank^2^ for comparative analysis of the eight *Lepidium LG*s with the five *A. thaliana* (*At*) chromosomes (*Atx-1*–*Atx-5*). All *LG*s had hits from multiple regions within the five *At* chromosomes, but to highly different extents (Table 2). This analysis revealed that the largest group of hits for *LG-8* was from *At* chromosome 5 (*Atx-5*) and covered 39% of *LG-8* sequences with mean sequence identity (*mSI*) of 82%, whereas the largest group of hits that covered 25% of *LG-5* with *mSI* of 85% came from *Atx-3*. These are composed of 11,383 (*LG-8*) and 9,820 (*LG-5*) matching sequences on *Atx-5* and *Atx-3*, respectively (Table 2). Similarly, the largest group of hits for *LG-1* (*mSI* = 92%), *LG-2* (*mSI* = 84%), *LG-3* (*mSI* = 91%), *LG-4* (*mSI* = 84%), *LG-6* (*mSI* = 84%), and *LG-7* (*mSI* = 82%) were from *Atx-1*, *Atx-1*, *Atx-3*, *Atx-2*, *Atx-4*, and *Atx-4*, respectively, and covered 21, 14, 26, 25, 10, and 18% of the corresponding *LG* sequences (Table 2). In the case of *LG-3* and *LG-6*, 11 and 9% of the sequences matched sequences of *Atx-2* and *Atx-5*, respectively. The sequence identity of the *LG*s with *At* chromosomes ranged from 80.6% (*LG-7*) to 85.0% (*LG-3*) with overall mean of 82.9%. Overall, 29, 28, 51, 41, 41, 26, 31, and 52% of *LG-1* to *LG-8* sequences, respectively, matched the sequences of *At* chromosomes (Table 2).

**FIGURE 6:**
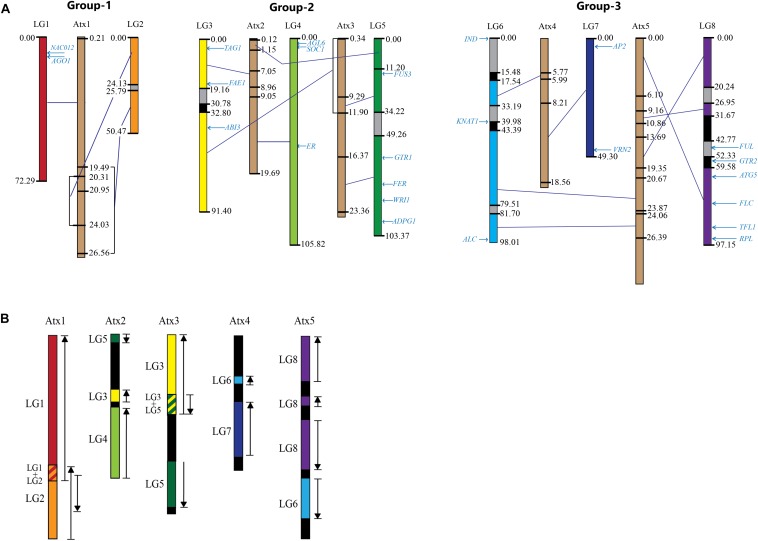
A comparison of the eight *Lepidium* linkage groups (*LG*s) and the five *A. thaliana* chromosomes (*Atx*s) forming three groups: **(A)** The *LG*s are shown in different colors whereas *Atx*s in brown; the numbers at the sides of the *LG*s and *Atx*s are map distance in centimorgan and sequence position in megabase pair, respectively, the text in light blue shows the map position of the homologs of genes known to regulate various traits in *A. thaliana*; gray and black regions within the *LG*s show regions lacking collinearity/matching sequences and mapped markers, respectively, deep blue lines connecting the *LG*s and *Atx*s show regions of high collinearity between them within the boundaries of the closest centimorgan (for *LG*s) and megabase pair (for *Atx*s); overlapping regions in collinearity are shown in square brackets. **(B)** The five *Atx*s displaying regions of collinearity with their corresponding *LG*s shown in different colors in **(A)**; upward arrows show collinearity in the same direction, whereas downward arrows show collinearity in opposite direction as a result of inversion.

The contigs/scaffolds mapped to *Lepidium LG*s were blast searched against the *At* genome, and the position of corresponding sequences within *At* chromosome sequences were determined. Based on major sequence coverage and identity, the correspondence between the *Lepidium LG*s and *At* chromosomes were grouped into three groups (Figure 6A). Group 1 shows the correspondence of *LG-1* and *LG-2* with *Atx-1*. Group 2 contains *LG-3*, *LG-4*, and *LG-5* as well as *Atx-2* and *Atx-3. LG-3* and *LG-5* mainly correspond to *Atx-3*, although *LG-3* also has a smaller portion that corresponds to *Atx-2*, whereas *LG-4* corresponds to *Atx-2*. Group 3 contains *LG-6*, *LG-7*, and *LG-8* as well as *Atx-4* and *Atx-5*. *LG-7* mainly correspond to *Atx-4*, whereas *LG-8* corresponds to *Atx-5*. On the other hand, *LG-6* has two major portions where one corresponds to *Atx-4* and the other to *Atx-5*. Blast searching of the sequences of the *LG*s against *At* genome revealed that the largest matching sequences between *LG-1*_*Atx-1*, *LG-2*_*Atx-1*, *LG-3*_*Atx3*, *LG-4*_*Atx-2*, *LG-5*_*Atx-3*, *LG-6*_*Atx-4*, *LG-6*_*Atx-5*, *LG-7*_*Atx-4*, and *LG-8*_*At-5* were 3.8, 3.7, 13.8, 6.5, 13.8, 11.1, 6.7, 14.1, and 12.8 kbp, respectively (Table 2).

The comparison of the *LG*s with *At* chromosomes revealed three regions within the *LG* sequences as shown in Figure 6A: (1) regions shown in colors other than gray and black showed high collinearity with *At* chromosome sequences, (2) regions shown in gray are either did not show significant collinearity or did not match sequences of *At* chromosomes, and (3) regions shown in black do not have mapped contigs and hence could not be compared with *At* chromosomes. In the highly collinear regions, collinearity is either in the same or opposite direction as compared to the corresponding *At* chromosome sequences, as shown in black upward and downward arrows, respectively, in Figure 6B. At least 66% of the sequences of the *LG*s showed high collinearity with *At* chromosome sequences. However, significant portions of *LG-3*, *LG-6*, and *LG-8* were either gray or black, whereas *LG-2* and *LG-5* have significant gray regions (Figure 6A). On the other hand, the whole regions of *LG-1*, *LG-4*, and *LG-7* showed high collinearity with their corresponding *At* chromosome sequences. *LG-2*, *LG-3*, *LG-5*, *LG-6*, and *LG-8* showed 92.7, 86.4, 85.2, 69.7, and 66.7% collinearity with their corresponding *At* chromosomes. On average, 87.6% of the *LG*s are collinear with the *At* chromosome sequences.

The map positions of the homologs of 24 *At* genes that are known to regulate various traits in *Arabidopsis* have been identified on *Lepidium LG*s (Table 3 and Figure 6A). *LG-1* carries the homologs of *NAC012* and *AGO1* genes on *Atx-1*. The homologs of *TAG1* (*Atx-2*), *ABI3* (*Atx-3*), and *FAE1* (*Atx-4*) were located on *LG-3*. *LG-4* carries the homologs of *AGL6*, *SOC1*, and *ER* genes on *Atx-2*. Similarly, the homologs of five genes on *Atx-3* (*FUSCA3*, *GTR1*, *FER*, *WRI1*, and *ADPG1*) were located on *LG-5*, whereas the homologs of *IND* and *KNAT1* (*Atx-4*) were located on *LG-6*, which also contained the homolog of *ALC* gene (*Atx-5*). *LG-7* carries the homologs of *AP2* and *VRN2* genes on *Atx-4*. The homologs of *FUL*, *GTR2*, *ATG5*, *FLC*, *TFL1*, and *RPL* genes that belong to *Atx-5* were located on *LG-8*. None of the homologs of the 24 *At* genes were located on *LG-2*.

**TABLE 3:** List of mapped scaffolds/contigs (Sc/Co) containing sequences of homologs of *Arabidopsis* genes regulating various traits.

***Arabidopsis thaliana***		***Lepidium***					
		**Sc/Co containing**		**Position of the**	**Gene sequence**	**No. of SNPs**	**Position of the**
**Gene**		**homolog of**	**Linkage**	**Sc/Co in the**	**portion in**	**in the Sc/Co**	**first and last**
**name**	**Chromosome**	**the gene**	**group**	**LG (cM)**	**the Sc/Co**		
**SNPs in the Sc/Co**							
*NAC012*	1	scaffold27952	1	14.35	1–1,106	0	–
*AGO1*	1	scaffold29006/scaffold7267/scaffold29563	1	14.35/15.45/15.45	1–2,474/3,674–3,890/1–1,700	0/1^*c*^/1^*d*^	–/3,742/122
*TAG1*	2	scaffold22710	3	3.75	11,738–11,987	0	–
*FAE1*	4	scaffold2900	3	19.16	10,838–3,988	1^*c*^	3301
*ABI3*	3	scaffold5739	3	40.40	21–2,277	0	–
*AGL6*	2	scaffold22580	4	2.20	4–542	0	–
*SOC1*	2	scaffold22580	4	2.20	2,437–5,736	0	–
*ER*	2	c1439524/scaffold34525	4	59.04	3–2,003/35,191–35,721	1^*c*^/1^*a*^	413/14,570
*FUSCA3*	3	scaffold34520	5	14.98–15.12	1–2,200	3^*a*^	59,689–11,892
*GTR1*	3	scaffold5096	5	66.30–67.26	152,442–155,021	6^*a*^	169,395–61,600
*FER*	3	scaffold5167	5	79.52–84.80	109,573–112,918	20^*a*^	61,206–365,527
*WRI1*	3	scaffold15935	5	89.86–90.73	84,778–88,945	13^*a*^	8,891–218,544
*ADPG1*	3	scaffold7650 (scaffold7649)	5	99.26	1,511–4,569	0	–
*IND*	4	scaffold27982	6	0.0–1.11	29,166–29,781	24^*a*^	210,061–271,934
*KNAT1*^*e*^	4	scaffold1526/scaffold28185	6	38.07/38.21	4,390–8,095/38,107–41,130	0	–
*ALC*	5	scaffold13810	6	96.08	3,292–4,327	0	–
*AP2*	4	scaffold31739/scaffold31828	7	3.40/6.45	91–2,006/91–2,035	0	–
*VRN2*	4	scaffold3823/scaffold3822	7	48.45/48.59	134–2,662/134–1,931	0	–
*FUL*	5	scaffold2744	8	42.77–45.69	3,336–12,103	6^*a*^	26,158–150,835
*GTR2*	5	c1485122	8	52.33	26,258–29,163	0	–
*ATG5*	3	scaffold17926	8	60.82	55,570–59,477	1^*a*^	115,257
*FLC*	5	scaffold34410	8	79.39–79.66	74,767–79,629	6^*a*^	32,287–73,222
*TFL1*	5	scaffold12832	8	88.94–94.26	109,716–110,791	26^*a*^	442,896–63,934
*RPL*	5	scaffold11723	8	96.08–96.61	116,530–119,818	5^*b*^	118,215–86,600

*^*a*^The SNP(s) is/are outside the gene sequence.^*b*^Two of the five SNPs (117224 and 118215) are within the coding sequence of the gene.^*c*^The SNP is in the coding sequence of the gene.^*d*^The SNP is within the gene sequence but outside the coding sequence.^*e*^The two scaffolds have overlapping sequence and hence refer to the same gene sequence. These scaffolds/contigs covered 38–99% of the full gene sequences with sequence identify varying from 73 to 87%. These scaffolds/contigs covered 33–100% of the coding sequences with sequence identify varying from 76 to 96%.*

## Discussion

Advanced next generation sequencing technologies allow identification of thousands of polymorphic markers that have various applications including the determination of genetic diversity and development of high-density genetic linkage map in plant species. The present study revealed an average observed heterozygosity (*Ho*) of <6% in both *L. campestre* and *L. heterophyllum* signifying that both species are predominantly inbreeders. In the F_2_ mapping population, only 13.1% of the filtered SNPs were heterozygous, on average, which is significantly lower than the 50% heterozygosity expected for the whole F_2_ population derived from the crosses of the two parental plants. Although random F_2_ seeds were planted, the seedlings of some of them were extremely weak or unhealthy at very young age, and consequently, leaf tissue was not sampled from such plants for DNA extraction. Given the obtained result of low proportion of heterozygous SNPs in the mapping population, it is likely that most of the unfit seedlings had higher proportion of heterozygosity across their genome. This finding may suggest that plants with higher proportion of heterozygosity across their genome perform poorly, the case that can be generally regarded as heterozygote disadvantage. This is in line with theoretical prediction that homozygosity is fixed easily in strongly selfing plant species if rearrangements reduce fitness (Charlesworth, 1992). The 51.9% heterozygosity for the 2,331 mapped SNPs is in line with the expected 50%, and this has been obtained through discarding SNPs deviating from 1:2:1 segregation with *P* < 0.01 to make it suitable for the linkage mapping.

Construction of linkage map is an important step in the identification of genes and molecular markers for its application in plant breeding. In this study, we used the GBS method (Baird et al., 2008; Elshire et al., 2011) to simultaneously discover new SNP markers and genotype individual samples from two *Lepidium* species (*L. campestre* and *L. heterophyllum*) as well as an F_2_ mapping population of interspecific hybrid between genotypes of the two species. SNP markers discovered through GBS has previously been successfully used for the construction of high-density genetic linkage mapping in various plant species including barley and wheat (Poland et al., 2012), *Aethionema arabicum* (Nguyen et al., 2019), *Avena* species (Latta et al., 2019), and grapevine (Tello et al., 2019). The eight linkage groups constructed in this study correspond to the eight haploid chromosome number previously reported for both *L. campestre* and *L. heterophyllum* (Rice et al., 2015).

The Brassicaceae family has been described as having four major evolutionary lineages (Bailey et al., 2006; Couvreur et al., 2010). The ancestral karyotype of lineages I and II is composed of eight chromosomes, which later evolved into the ancestral Camelineae karyotype of eight chromosomes and the Calepineae karyotype of seven chromosomes (Murat et al., 2015). *Lepidium* and *Arabidopsis* belong to lineage 1 and evolved from ancestral Camelineae karyotype of eight chromosomes, which makes it suitable to transfer genomic information from *Arabidopsis* (a model species) to *Lepidium* (species of agronomic interest). Hence, the sequences of *A. thaliana* chromosomes were used as a tool for comparative analyses of *Lepidium* and *Arabidopsis* genomes in this study.

The *LG*s of *L. campestre* are named from *LG-1* to *LG-8* in a way that they match previously reported chromosome nomenclature of Brassicaeae species with a haploid chromosome number of eight (*n* = 8), such as *Arabidposis lyrata* (Boivin et al., 2004; Kuittinen et al., 2004; Yogeeswaran et al., 2005; Hu et al., 2011; Murat et al., 2015). *LG-1* to *LG-8* match chromosomes 1–8 of *A. lyrata* in that order. Studies on different Brassicaceae species have revealed chromosomal events through which a karyotype of *n* = 8 in an ancestral species shared by *Lepidium* and *Arabidopsis* have evolved to a karyotype of *n* = 5 in *A. thaliana* (Boivin et al., 2004; Kuittinen et al., 2004; Yogeeswaran et al., 2005; Koch and Kiefer, 2005; Lysak et al., 2006; Hu et al., 2011; Murat et al., 2015). The comparative genomic analysis between *A. lyrata* (*n* = 8) and *A. thaliana* (*n* = 5) revealed that more than 50% the *A. lyrata* genome is absent in *A. thaliana*, whereas ∼25% the *A. thaliana* genome is missing in *A. lyrata* (Hu et al., 2011), which is the result of accumulated chromosomal and point mutations since the two species separated roughly 5–6 million years ago (mya) (Koch and Kiefer, 2005). However, the overall sequence identity between their homologous sequences is >80% (Hu et al., 2011), which is comparable with the overall sequence identity of 82.9% between homologous sequences of *L. campestre* and *A. thaliana*.

The degree to which genes and genomic regions are maintained on corresponding chromosomes (remain syntenic) and in corresponding orders (remain collinear) over a period of time varies among eukaryotic genomes (Coghlan et al., 2005). Correlating arrangements of genomic regions of a model species with a related species allows inference of shared ancestry of genes as well as utilization of known genetic information of the model species to study less-well-understood systems (Tang et al., 2008). About 10 major rearrangements have been reported between *A. thaliana* and *A. lyrata*, including two reciprocal translocations and three chromosomal fusions (Kuittinen et al., 2004; Yogeeswaran et al., 2005; Lysak et al., 2006) that resulted in the formation of a karyotype of five chromosomes in *A. thaliana* from the ancestral state of eight chromosomes that still exist in other Brassicaceae species, such as *A. lyrata*. The fusions and translocations reported based on the karyotypes of the two *Arabidopsis* species are also evident in this study through comparison of the *L. campestre* and *A. thaliana* genomes. As a result of the fusion of ancestral chromosomes, *LG-1* and *LG-2* matched *Atx-1*, *LG-3*, and *LG-4* matched *Atx-2*, and *LG-8* and *LG-6* matched *Atx-5*. In all three pairs, the first *LG*s matched the upper part of their corresponding *At* chromosomes. *LG-5* corresponds to *Atx-3*, whereas *LG-7* corresponds to *Atx-4*.

The inversion within the lower arm of *Atx-1* that corresponds to ancestral chromosome-2 (Lysak et al., 2006) was also evident in this study (inversion of corresponding region of *LG-2*). Following the fusion of ancestral chromosomes 3 and 4, unequal reciprocal translocation occurred between the fused chromosome (at region corresponding to chromosome 3) and the upper part of ancestral chromosome 5 (Lysak et al., 2006; Hu et al., 2011), which is in line with the present study (Figures 6A,B). However, unlike the case between *A. lyrata* and *A. thaliana*, the comparison of *Lepidium* and *Arabidopsis* genomes revealed inversion of the translocated block to the fused chromosome, suggesting the occurrence of further major chromosomal rearrangements after the *Arabidopsis* and *Lepidium* lineages were separated. Following the fusion of ancestral chromosomes 6 and 8, unequal reciprocal translocation occurred between the fused chromosome (at region corresponding to chromosome 6) and the upper part of ancestral chromosome 7 (Lysak et al., 2006; Hu et al., 2011), which is in agreement with the present study (Figures 6A,B). Among the two inversions previously reported (Lysak et al., 2006; Hu et al., 2011), the inversion within *Atx-5* was evident but not the one within *Atx-4*.

Parkin et al. (2005) identified 21 shared syntenic blocks between *A. thaliana* and *Brassica napus* genomes representing collinear regions maintained since the divergence of their lineages approximately 20 mya. The genomes of *A. thaliana* and *A. lyrata* are ∼90% syntenic that predominately showed highly conserved collinear arrangements, although multiple inversions also exist between the genomes (Hu et al., 2011). Similarly, the present study showed an average of 87.6% synteny between the sequences of *Lepidium LG*s and *A. thaliana* chromosomes, and inversion of small segments are common throughout the *Lepidium LG*s. Except in *LG-1*, *LG-4*, and *LG-7*, the other *LG*s have significant regions that are unalignable with *A. thaliana* chromosomes similar to the case between *A. thaliana* and *A. lyrata* that have unalignable regions throughout the genome. The latter case is mainly due to deletions throughout *A. thaliana* genome, suggesting that deletions are favored over insertions and hence smaller genome (Hu et al., 2011).

Through comparative analysis of genomic sequences, the linkage map positions of the homologs of 24 genes that are known to regulate various traits in *A. thaliana* have been located on *Lepidium LG*s (Table 3 and Figure 6A). These genes regulate traits that are targeted for improvement within the domestication project of *L. campestre*. The homologs of the *NAC DOMAIN CONTAINING PROTEIN 12* (*NAC012*) and *ARGONAUTE* 1(*AGO1*) were mapped to *LG-1*. *NAC012* contributes to the regulation of pod shattering in *A. thaliana* through controlling the development of secondary walls in siliques (Rajani and Sundaresan, 2001; Liljegren et al., 2004). The sequence identity between the *NAC012* partial coding sequences of these two species was 92% (Gustafsson et al., 2018). *AGO1* is involved in the determination of inflorescence architecture in *Arabidopsis* through suppressing the *TERMINAL FLOWER 1* (*TFL1*) (Ferrándiz-Nohales et al., 2014).

The homologs of *TRIACYLGLYCEROL BIOSYNTHESIS DEFECT 1* (*TAG1*), *ABA-insensitive 3* (*ABI3*), and *FATTY ACID ELONGATION* 1 (*FAE1*) were mapped to *LG-3*. *TAG1* is one of the genes involved in the biosynthesis of fatty acids, and it regulates oil production (Routaboul et al., 1999; Jako et al., 2001); *FAE1* controls seed oil composition (James et al., 1995), and *ABI3* regulates seed dormancy (Ooms et al., 1993) in *Arabidopsis*. The sequence identity of *TAG1* and *FAE1* between the partial coding sequences of these two species was 93 and 88%, respectively (Gustafsson et al., 2018). The homologs of *A. thaliana AGAMOUS LIKE 6* (*AGL6*), *SUPRESSOR OF OVEREXPRESSION OF CO1* (*SOC1*), and *ERECTA* (*ER*) were mapped to *LG-4*. *AGL6* and *SOC1* are flowering time genes that regulate flowering in *Arabidopsis*. The sequence identity of *AGL6* and *SOC1* between the partial coding sequences of these two species was 98 and 94%, respectively (Gustafsson et al., 2018). *ER* regulates traits such as internode length and angles of pods (Douglas et al., 2002; Venglat et al., 2002), which have direct effect on seed yield through the determination of the number of pods on each inflorescence.

The homologs of FUSCA 3 (FUS3), GLUCOSINOLATE TRANSPORTER-1 (GTR1), FERONIA (FER), WRINKLED (WRI1), and ARABIDOPSIS DEHISCENCE ZONE POLYGALACTURONASE 1 (ADPG1) were mapped to LG-5. FUS3 regulates seed dormancy (Luerssen et al., 1998), whereas GTR1 regulates glucosinolates transport (Andersen and Halkier, 2014; Saito et al., 2015) in Arabidopsis. FER is a host-plant resistance gene (Kessler et al., 2010), WRI1 regulates oil production (Focks and Benning, 1998), and ADPG1 is one of the genes regulating fruit dehiscence (Ogawa et al., 2009) in Arabidopsis. A. thaliana and L. campestre showed sequence identity of 93, 88, and 91% for coding region partial sequences of FER, WRI1, and ADPG1, respectively (Gustafsson et al., 2018). The homologs of INDEHISCENT (IND), KNOTTED-LIKE FROM ARABIDOPSIS THALIANA-1 (KNAT1), and ALCATRAZ (ALC) were mapped to LG-6. IND and ALC are valve identity genes responsible for the establishment of the valve margin in the seed-containing pod and thereby regulate pod shattering in Arabidopsis (Rajani and Sundaresan, 2001; Liljegren et al., 2004). KNAT1 is a gene that determines pod density through regulating traits such as internode length and angles of pods (Douglas et al., 2002; Venglat et al., 2002). The alignment of A. thaliana and L. campestre partial coding sequences showed 85 and 83% sequence identity for IND and ALC genes, respectively (Gustafsson et al., 2018).

The homologs of *APETALA2* (*AP2*) and *VERNALIZATION 2* (*VRN2*) were mapped to *LG-7*. *AP2* is a transcription factor gene involved in the regulation of flowering and seed development (Okamuro et al., 1997) as well as controlling seed yield (Jofuku et al., 2005), whereas *VRN2* regulates vernalization responses (Gendall et al., 2001) in *Arabidopsis*. *AP2* and *VRN2* showed 89 and 90% sequence identity, respectively, between *A. thaliana* and *L. campestre* in their partial coding sequences (Gustafsson et al., 2018). The homologs of *FRUITFULL* (*FUL*), *GLUCOSINOLATE TRANSPORTER-2* (*GTR2*), *AUTOPHAGY RELATED 5* (*ATG5*), *FLOWERING LOCUS C* (*FLC*), *TERMINAL FLOWER 1* (*TFL1*), and *REPLUMLESS* (*RPL*) were mapped to *LG-8*. FUL controls the development of the wall of seedpods (valve) (Gu et al., 1998; Ferrándiz et al., 2000) and thereby regulates pod shattering. *GTR2* regulates glucosinolates transport (Andersen and Halkier, 2014), whereas *ATG5* is a gene involved in plant defense (Yoshimoto et al., 2009). *FLC* is a MADS-box gene that plays a key role in regulating plant developmental responses to temperature as well as flowering (Michaels and Amasino, 1999; Sheldon et al., 2000). *TFL1* is a gene involved in the determination of inflorescence architecture in *Arabidopsis* (Ferrándiz-Nohales et al., 2014), whereas *RPL* is one of several genes responsible for the establishment of the valve margin in the seed-containing pod and thereby involved in the regulation of pod shattering (Roeder et al., 2003). According to Gustafsson et al. (2018), the partial coding sequences of *FUL*, *GTR2*, *ATG5*, *FLC*, and *RPL* showed 92, 88, 81, 90, and 89% sequence identity, in that order, between *A. thaliana* and *L. campestre*.

In summary, the eight *LG*s revealed in this study confirm the previously reported (1) haploid chromosome number of eight in *L. campestre* and *L. heterophyllum*; (2) chromosomal fusion, translocation, and inversion events during the evolution of *n* = 8 karyotype in ancestral species shared by *Lepidium* and *Arabidopsis* to *n* = 5 karyotype in *A. thaliana*. The construction of high-density genetic linkage map bearing thousands of polymorphic markers and the identification of homologs of various desirable genes in the present study are significant steps toward the application of marker-aided and genomic selection in *L. campestre* for its accelerated domestication.

## Data Availability Statement

The datasets generated for this study can be found in the DDBJ/ENA/GenBank under the accession WJSH00000000. The version described in this paper is version WJSH01000000.

## Author Contributions

RO and MG secured the funding. CG and MG developed the mapping population and extracted DNA and conducted comparative genomics analysis. JG generated the GBS data and constructed genetic linkage map. MG and JG wrote the manuscript. All authors conceived and designed the study, contributed to data analysis, revised the manuscript, and read and approved the final version of the manuscript for publication.

## Conflict of Interest

The authors declare that the research was conducted in the absence of any commercial or financial relationships that could be construed as a potential conflict of interest.

## Footnotes

1 https://blast.ncbi.nlm.nih.gov/Blast.cgi

2 https://www.ncbi.nlm.nih.gov/genbank/

## Supplementary Material

FIGURE S1 A framework genetic map comprising 2331 SNP markers on eight linkage groups: The rows are F_2_ individuals and the columns (each only a few pixels wide) are markers; the vertical black lines separate the linkage groups; blue is heterozygous, green is homozygous for parent-1, yellow is homozygous for parent-2, and pink is missing data.

TABLE S1 **(A)** list of the 2330 mapped SNPs on 1044 contigs/scaffolds, **(B)** final ordering of the 10302 genetically mapped contigs/scaffolds, **(C)** List of contigs/scaffolds with mapped SNPs for each linkage group arranged according to the number of mapped SNPs, **(D)** summary of data provided in **(A)**, **(B)** and **(C)**.

TABLE S2 **(A)** List of contigs/scaffolds mapped by SNPs, and **(B)** cumulative percentage of contigs with >1 mapped SNPs.

TABLE S3 **(A)** List of the 34342 genetically mapped tags from contigs, and **(B)** list of the 9931 mapped contig tag haplotypes.
