# Supplementary figures and images for: High-Density Genetic Linkage Mapping of Lepidium Based on Genotyping-by-Sequencing SNPs and Segregating Contig Tag Haplotypes
Source: Front Plant Sci. 2020 Apr 30;11:448. doi: 10.3389/fpls.2020.00448 (PMC7204607; doi:10.3389/fpls.2020.00448)

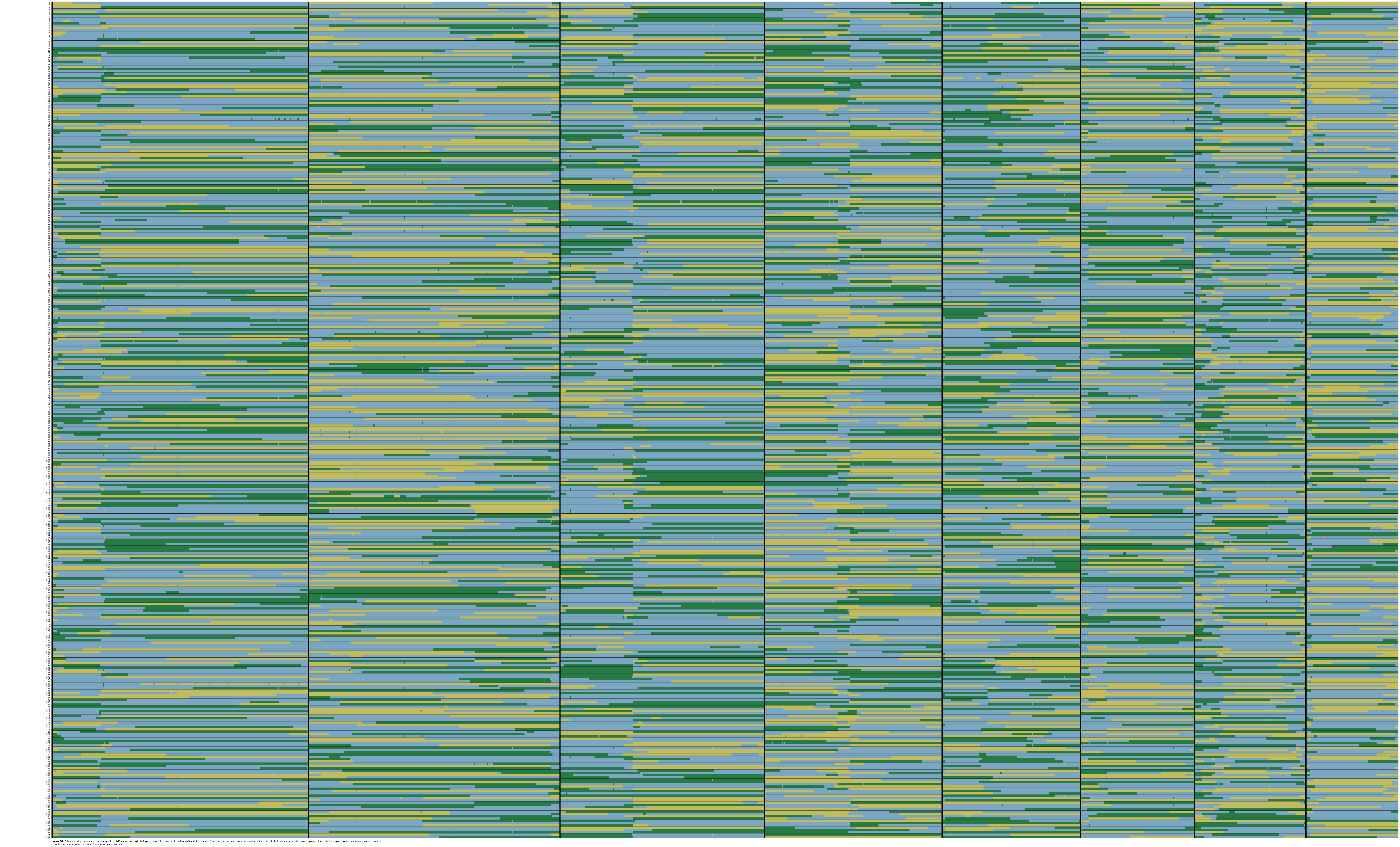

Supplement: FIGURE S1 — A framework genetic map comprising 2331 SNP markers on eight linkage groups: The rows are F2 individuals and the columns (each only a few pixels wide) are markers; the vertical black lines separate the linkage groups; blue is heterozygous, green is homozygous for parent-1, yellow is homozygous for parent-2, and pink is missing data. [file Image_1.TIFF]
